# Whole-Exome Sequencing Reveals New Potential Mutations Genes for Primary Mucosa-Associated Lymphoid Tissue Lymphoma Arising From the Kidney

**DOI:** 10.3389/fonc.2020.609839

**Published:** 2021-01-08

**Authors:** Shuang Wen, Tianqing Liu, Hongshuo Zhang, Xu Zhou, Huidan Jin, Man Sun, Zhifei Yun, Hong Luo, Ze Ni, Rui Zhao, Bo Fan

**Affiliations:** ^1^ Department of Pathology, Dalian Friendship Hospital, Dalian, China; ^2^ Department of Biochemistry, Institute of Glycobiology, Dalian Medical University, Dalian, China; ^3^ Section of Experimental Surgery, Heidelberg University Hospital, Heidelberg, Germany; ^4^ Department of Anaesthesiology, Fifth Affiliated Hospital of Sun Yat-Sen University, Zhuhai, China; ^5^ Department of Clinical Medicine, Second Affiliated Hospital of Dalian Medical University, Dalian, China; ^6^ Department of Pharmacy, Zhongshan College of Dalian Medical University, Dalian, China; ^7^ Department of Urology, Second Affiliated Hospital of Dalian Medical University, Dalian, China

**Keywords:** mucosa-associated lymphoid tissue lymphoma, kidney, whole-exome sequencing, prognosis, therapy

## Abstract

Low-grade B cell lymphomas of mucosa-associated lymphoid tissue (MALT) lymphomas involving the kidney were extremely rare, genetic alteration or molecular features was not yet explored, which may lead to limited choices for postoperative adjuvant or targeted. Whole-exome sequencing based tumor mutation profiling was performed on the tumor sample from a 77-year-old female presenting with discomfort at the waist was pathologically diagnosed as MALT lymphomas in the right kidney. We identified 101 somatic SNVs, and the majority of the identified SNVs were located in CDS and intronic regions. A total of 190 gain counts of CNVs with a total size of 488,744,073 was also investigated. After filtering with the CGC database, seven predisposing genes (ARID4A, COL2A1, FANCL, ABL2, HSP90AB1, FANCA, and DIS3) were found in renal MALT specimen. Furthermore, we compared somatic variation with known driver genes and validated three mutational driver genes including ACSL3, PHOX2B, and ADCY1. Sanger sequencing of germline DNA revealed the presence of a mutant base T of PHOX2B and a mutant base C of ADCY1 in the sequence, which were discovered for the first time in MALT lymphomas involving the kidney. Moreover, immunohistochemical analysis revealed that tumor cells were positive for CD20, CD79a, PAX5, CD21, and CD23, and expression of CD3, CD5, and CD8 were observed in reactive T lymphocytes surrounding tumor cells. These findings illustrated that concurrent aberrant PHOX2B and ADCY1 signaling may be a catastrophic event resulting in disease progression and inhibition of the putative driver mutations may be alternative adjuvant therapy for MALT lymphoma in the kidney which warrants further clinical investigation.

## Introduction

Primary renal lymphomas usually had high-grade diffuse large B cell lymphomas as the main type, while low-grade mucosa-associated lymphoid tissue (MALT) lymphomas are rare. MALT lymphoma is a unique type of extranodal non-Hodgkin’s lymphoma. It is a type of B cell lymphoma in the marginal region and has a unique biological behavior, histology, and immunophenotype (1–3). In 1991, Pelsdving and colleagues first reported a case of primary MALT lymphoma in the kidney (4). At present, the diagnosis of primary MALT lymphoma in the kidney mainly relies on a pathological examination. The majority of specimens are derived from kidneys that are clinically suspected of renal cell carcinoma and are surgically removed, and a small portion is derived from biopsies of tumor puncture (5–7). Tumor cells initially surround the reactive follicles, infiltrate the outside of the follicular cannula, distribute in the marginal zone, and gradually diffuse to form a large area of fusion, eventually encroaching on some or all of the follicles. Typical marginal B cells are small- to medium-sized cells with mildly irregular nuclei, moderate chromatin, and indistinct nucleoli, similar to the central cells, and relatively abundant and lightly cytoplasmic. When cytoplasms are lightly stained, they can have a monocyte-like or small lymphocyte appearance. Approximately one-third of cases may have plasma cell differentiation (8–11). Transformed centroblasts or immunoblast-like macrophages can be seen in tumors, but these cells are not numerous. Residual glomeruli and tubules can be seen in the tumor. Tumor cells invade the glomerulus, renal tubules, or renal pelvis epithelial tissue and form lymphoepithelial lesions.

Although related literature for cases had been reported after searching the PubMed, Ovid MEDLINE, Google Scholar and Scopus databases, genetic characteristics or molecular features about primary MALT lymphoma in the kidney had not been investigated so far. Meanwhile, clinical parameters of primary MALT lymphoma in the kidney had not described systematically. As Next-Generation Sequencing (NGS) provides strong support for the detection of genetic alterations in tumors, herein, we performed whole-exome sequencing for a 77-year-old female presenting with discomfort at the waist which was pathologically diagnosed as MALT lymphomas in the right kidney. While identifying SNPs and INDEs, Somatic SNVs, INDELs and CNVs between primary renal MALT specimen and adjacent tissue specimen, we also found novel ACSL3, PHOX2B, and ADCY1 mutation which were driver activating mutation. Additionally, immunohistochemistry for markers of B lymphocyte or non-germinal center lymphocyte (CD20, CD79a, PAX5), markers of reactive T lymphocytes surrounding tumor cell of MALT (CD3, CD5, CD8) and indicators for differential diagnosis of primary renal MALT lymphoma and other blood system tumors (CD21, CD23, BCL-6, and CD10) contributed to explore abnormal changes in terms of molecular pathology. Finally, we also collected information by reviewing related literature to explore patient-specific characteristics, tumor-specific features, possible prognoses and therapeutic strategies.

## Materials and Methods

### Clinical Analysis

The relevant clinical information was retrieved from Dalian Friendship Hospital. Institutional review board approval from Dalian Friendship Hospital was granted.

### Immunohistochemistry

Specimens were fixed in 10% formalin, embedded in paraffin (4 μm thickness), stained with hematoxylin and eosin (HE), and visualized with light microscopy. IHC was performed on formalin-fixed paraffin-embedded (FFPE) tissue sections and we also selected normal tonsil tissue that had abundant lymphoid tissues as a positive control for IHC. After dewaxing in xylene overnight and rehydration in a gradient of alcohol solutions, the sections were heated in an antigen retrieval buffer at 120°C for 6 s. When endogenous peroxidases were blocked by 3% hydrogen peroxide for 30 s, the sections were incubated with several antibodies at 4°C for 24 as follows: rabbit polyclonal anti-Ki67 (dilution 1:6000, 27309-1-AP, ProteinTech Group, Chicago, USA), rabbit monoclonal anti-CD20 (dilution 1:100, ab78237, Abcam, Cambridge, UK), rabbit monoclonal anti-CD79a (dilution 1:100, ab79414, Abcam), rabbit monoclonal anti-PAX5 (dilution 1:1000, ab109443, Abcam), rabbit polyclonal anti-BCL-2 (dilution 1:200, 12789-1-AP, ProteinTech Group), rabbit monoclonal anti-CD3 (dilution 1:100, ab16669, Abcam), Rabbit Polyclonal anti-CD5 (dilution 1:800, 17227-1-AP, ProteinTech Group), mouse monoclonal anti-CD8 (dilution 1:6000, 66868-1-Ig, ProteinTech Group), rabbit monoclonal anti-CD21 (dilution 1:300, ab75985, Abcam), rabbit monoclonal anti-CD23 (dilution 1:100, ab92495, Abcam), rabbit polyclonal anti-BCL-6 (dilution 1:400, 21187-1-AP, ProteinTech Group), rabbit monoclonal anti-CD10 (dilution 1:500, ab256494, Abcam), rabbit monoclonal anti-CyclinD1 (dilution 1:100, ab16663, Abcam). The samples were then incubated with biotin-labeled secondary antibodies and avidin-labeled horseradish peroxidase and developed using DAB. The stromal expression level was evaluated by two investigators (Shuang Wen and Tianqing Liu) who were blinded to the clinical and prognostic information.

### DNA Extraction

Genomic DNA of primary renal MALT specimen and adjacent or surrounding normal tissue were extracted from the FFPE materials using the GeneRead DNA FFPE Kit (Qiagen, Germany) according to the manufacturer’s protocol. The quality of isolated genomic DNA was examined by using 2 methods: 1% agarose gel electrophoresis to monitor DNA degradation and contamination and a Qubit^®^ DNA Assay Kit on a Qubit^®^ 2.0 Fluorometer (Invitrogen, USA) to detect DNA concentration.

### Library Preparation and Sequencing

For whole-exome sequencing, genomic DNA was fragmented into segments of 180–280 bp by a Covaris instrument (Covaris, Massachusetts, USA). The whole-exome was captured using an Agilent SureSelect Human All Exon Kit (Agilent Technologies, CA, USA) following the manufacturer’s recommendations. The products were purified using the AMPure XP system (Beckman Coulter, Beverly, USA) and quantified using the Agilent high sensitivity DNA assay on the Agilent Bioanalyzer 2100 system. DNA libraries with average insert sizes of 150 bp were sequenced at the Novogene sequencing facility using an Illumina HiSeq platform (Illumina, San Diego, CA, USA).

### Quality Control

Raw data obtained by sequencing contain a small number of adapter reads, low-quality nucleotides, and undetected nucleotides. To ensure the quality of the analysis, it is necessary to filter raw reads to obtain clean reads, and subsequent analyses will be conducted on the basis of clean reads. Paired reads containing adapter contamination, reads in which more than 10% of bases were uncertain, and reads in which the proportion of low-quality (Phred quality <5) bases was over 50% were removed. High-quality clean data were obtained and applied to guarantee a meaningful downstream analysis.

### Bioinformatics Analysis

Valid sequencing reads were aligned to the reference genome (UCSC hg19) using Burrows-Wheeler Aligner (BWA) software to produce BAM files. Then, the BAM files were sorted by SAMtools, and duplicate reads were marked by Picard (http://broadinstitute.github.io/picard/). Finally, the final BAM file was used to compute the sequence coverage and depth. We used Picard to mark these duplicates for a follow-up analysis.

### Variant Calling, Functional Annotation, and Somatic Mutation Calling

We identified and filtered variants (single nucleotide polymorphisms (SNPs) and insertion and deletions (INDELs)) using mpileup and bcftools in SAMtools to guarantee a meaningful analysis. Variant call formats (VCFs) obtained from previous steps were characterized based on dbSNP and 1000 Genomes databases and other related existing databases and annotated with ANNOVAR. Somatic single nucleotide variants (SNVs) were detected by MuTect, and somatic INDELs were detected by Strelka. Control-FREEC was used to detect somatic copy number variations (CNVs).

### Identification of Potential Driver Mutations and Predisposing Genes

To identify potential driver mutations that are associated with the progression of carcinogenesis, we compared sample mutations to known driver mutations with inhouse software. Four driver mutation databases, Cancer Gene Census (CGC) 513, Bert Vogelstein 125, SMG 127, and Comprehensive 435, were utilized to analyze potential driver mutations for each sample group. Moreover, after exploring germline mutations (SNVs, INDELs) in normal tissues from patients with SAMtools software, potential predisposing genes were identified by detecting germline mutations and comparing them with the CGC (http://cancer/sanger.ac.uk/cancergenome/projects/census) database and two susceptibility gene databases with inhouse software.

## Results

### Case Presentation

**Figure 1:**
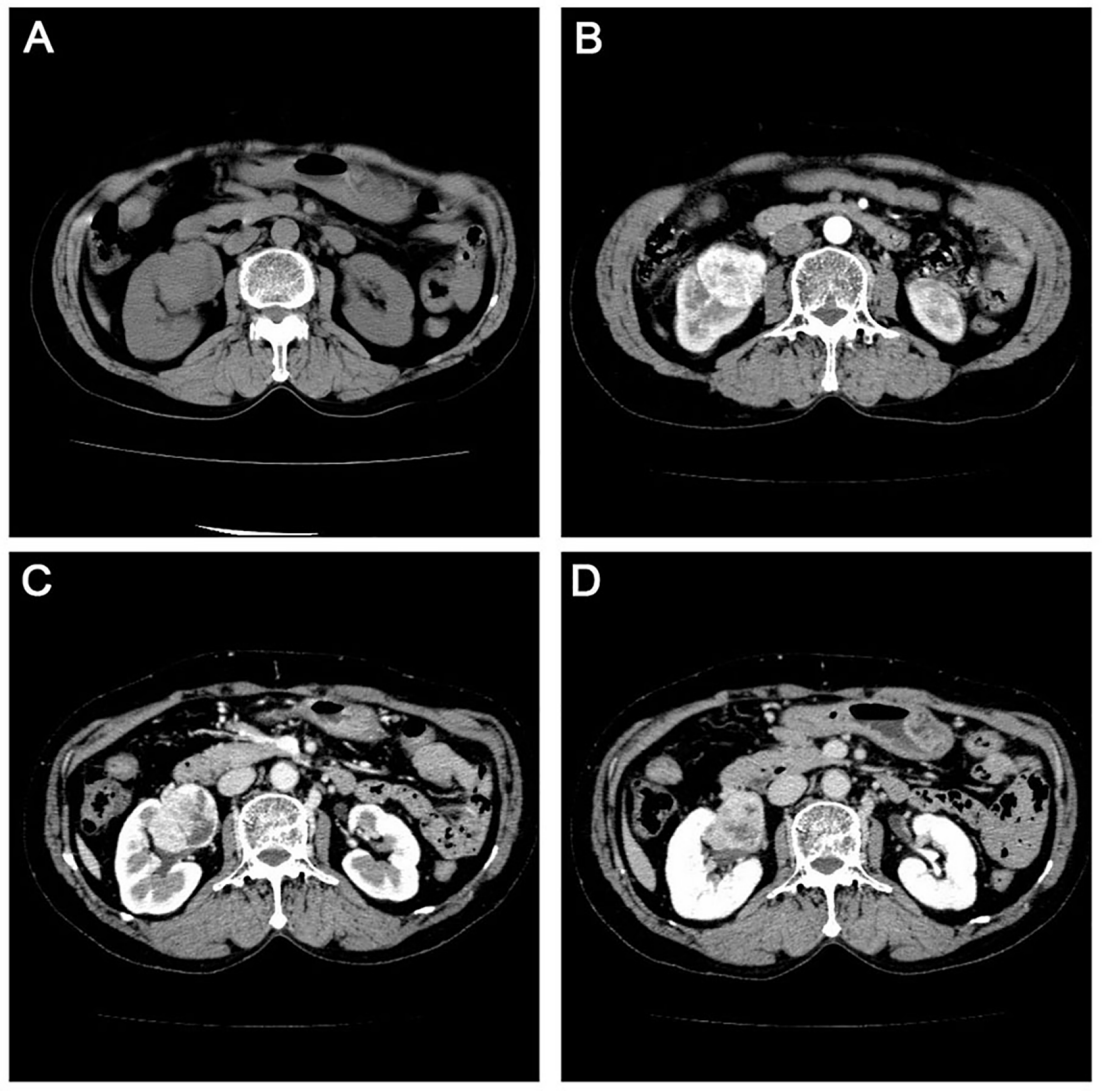
Computerized tomography scan in the urinary system with flat sweep **(A)** enhanced cortical phase. **(B)** enhanced medulla phase. **(C)** and enhanced excretion period **(D)** showing an enlarged right kidney measuring 12.3 cm x 6.6 cm. There was no echoic area around the kidney, and the larger area was located in the upper pole.

A 77-year-old female was referred to Dalian Friendship Hospital in October 2016 with discomfort at the waist as the main complaint. Urinary system ultrasound demonstrated a larger area mass measuring 7.1x6.2 cm in the upper pole of the right kidney. Abdominal computed tomography (CT) showed diffuse lesions of the right kidney without enlarged lymph nodes in the kidney or at other abdominal sites (**Figure 1**). Laboratory result of HCV, HBV, HIV, monoclonal component or autoimmune autoantibodies as follows: HBsAg 0.01 IU/ml (reference range 0.0-0.05 IU/ml); HBsAb 4.68 MIU/ml (reference range 0.0–10.0 MIU/ml); HBeAg 0.31 s/co (reference range 0.0–1.0 s/co); HBeAb 1.70 s/co (reference range > 1.0 s/co); HBcAb 3.31 s/co (reference range 0.0–10.0 s/co); HCV (-) 0.03 col (reference range <1 col); HIV (-); Antinuclear antibody (-); IgG of anti-double-stranded DNA antibody 12 IU/ml (reference range <24 IU/ml); Anti-neutrophil cytoplasmic antibody (-); Anti-cyclic cucurbita polypeptide antibody 2.9 U/ml (reference range <5 U/ml); Anti-keratin antibody (-); Anti-centromere antibody (-); Anti-cardiolipin antibody (-). As the clinical diagnosis was right kidney rupture bleeding, and the possibility of renal cell carcinoma was not excluded, nephrectomy for the right kidney was performed. The patient was pathologically diagnosed with primary MALT lymphoma arising from the kidney. As Ann Arbor stage was IE stage and MALT- international prognostic index score was 1 score which indicated that the patient was at low risk group, no additional treatment was given. During our follow-up, the patient had no evidence of disease 49 months after surgery.

### Histopathological Considerations

**Figure 2:**
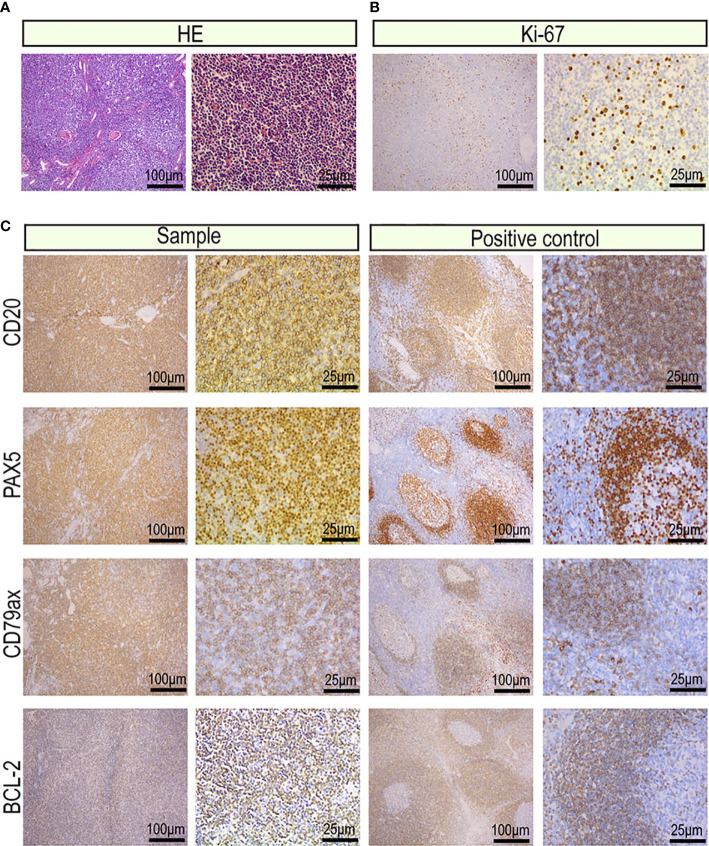
Images of the tumor cells from the primary renal MALT lymphoma (scale bar: 100μm, 25μm). **(A)** Hematoxylin and eosin-stained tumor sections. **(B)** Representative IHC images for Ki-67 proliferation index of 20%–25%. **(C)** Representative IHC images for CD20, CD79a, PAX5 which were markers of B lymphocyte and BCL-2 which was a marker of non-germinal center lymphocyte.

Visual examination for resection specimen of right kidney measured 12.8 cm x 8.0 cm x 5.5 cm. The renal capsule was thickened and separated from the parenchyma, with old bleeding underneath. The tumor measured 6.0 cm x 5.5 cm x 4.2 cm was observed in the parenchyma of the kidney. The mass and the surrounding kidney tissue had unclear boundaries, and the cut surface was gray, fine, and soft. Under microscopic examination, the normal kidney tissue in the tumor was replaced by a follicular-like structure, and the follicular hyperplasia was irregular. The marginal area of the follicles widened and expanded, and local fusion occurred. The marginal area was composed of diffuse uniform tumor cells, small to medium-sized lymphocytes, central cell-like cells, small lymphoid cells, and focal mononuclear B cells (Shown in **Figure 2A**). The central cell-like cells exhibited slightly irregular nuclei, abundant cytoplasms, light staining, moderate chromatin, and indistinct nucleoli, similar to the central cells. The mononuclear B cells were abundant and transparent in the cytoplasm. The nucleus was round or oval, and the chromatin was evenly distributed. Tumor cells were implanted into lymphoid follicles, causing partial or total destruction. Residual glomeruli and tubules were visible in the tumor, and lymphoepithelial lesions were observed.

### Immunohistochemical Profile

**Figure 3:**
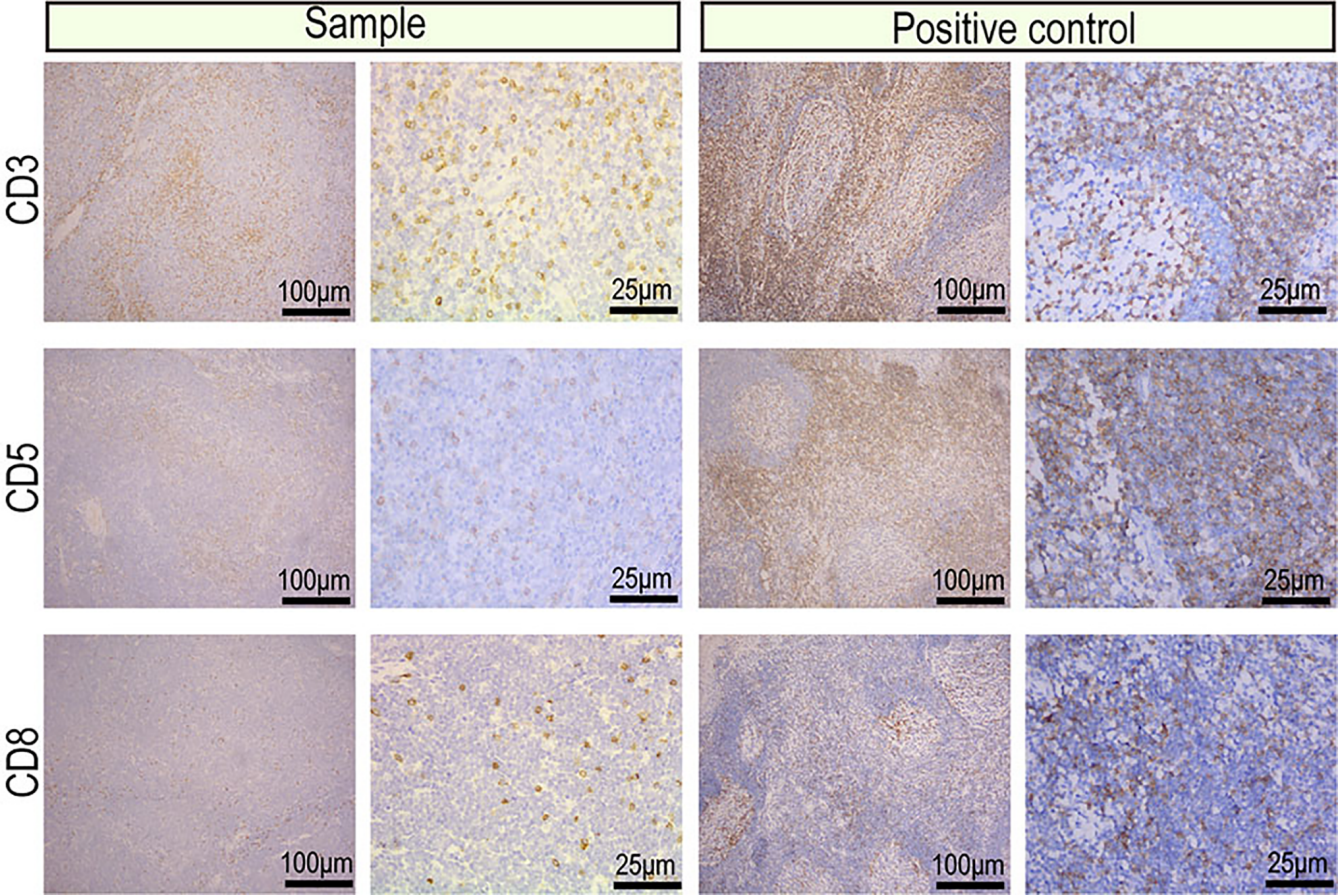
Images of the reactive T cells surrounding tumor cells from the primary renal MALT lymphoma (scale bar: 100μm, 25μm). Representative IHC images for CD3, CD5, and CD8 which were markers of reactive T cells.

**Figure 4:**
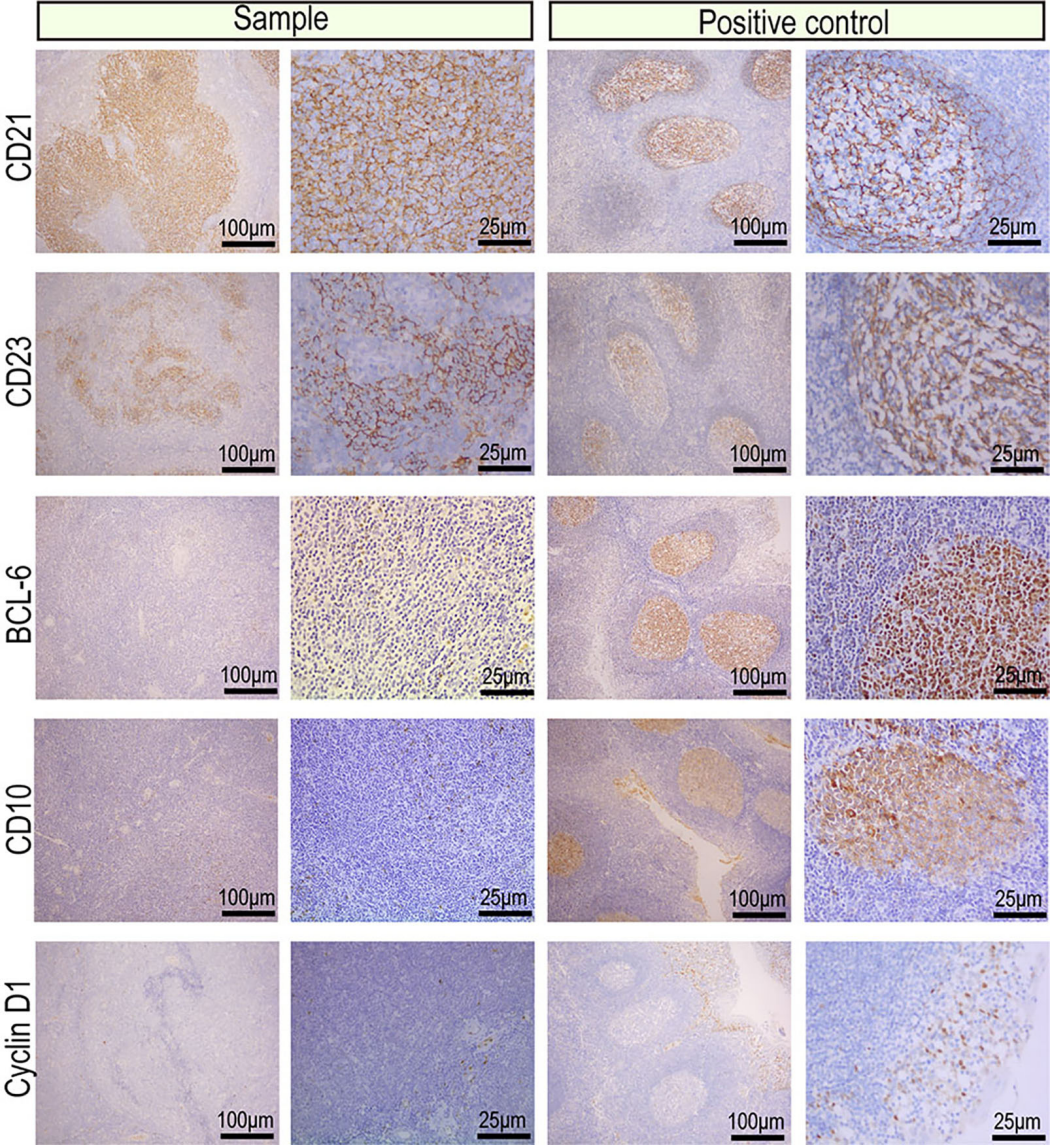
IHC images of the indicators for differential diagnosis of primary renal MALT lymphoma and other blood system tumors. Representative IHC images for CD21 and CD23 showed an irregular and damaged follicular dendritic network which was related to lymphoma. A negative representative for markers of germinal center lymphocyte, BCL-6 and CD10 may help differentiate from follicular lymphoma. Moreover, a negative representative of CyclinD1 may distinguish between mantle cell lymphoma or partial plasmablastic lymphoma and primary renal MALT lymphoma.

The protein expression profile by IHC was consistent in cancer manifestation of primary renal MALT. The Ki-67 proliferation index was 20%, indicating that cell proliferation activity was not high (**Figure 2B**). CD20, CD79a, PAX5 which were markers of B lymphocyte was strong in almost 90% of tumor nuclei (**Figure 2C**). Expression of CD3, CD5, and CD8 was observed in reactive T lymphocytes surrounding tumor cells and not in tumor nuclei (**Figure 3**). To differential diagnosis of primary renal MALT lymphoma and other blood system tumors in **Figure 4**, nuclear CD21 and CD23 expression in tumor cell showed irregular and damaged follicular dendritic network which was related to lymphoma. A negative representative for markers of germinal center lymphocyte, BCL-6, and CD10 may help differentiate from follicular lymphoma. Moreover, cyclinD1 staining may distinguish between mantle cell lymphoma or partial plasmablastic lymphoma and primary renal MALT lymphoma.

### Identification of SNPs and INDELs

**Figure 5:**
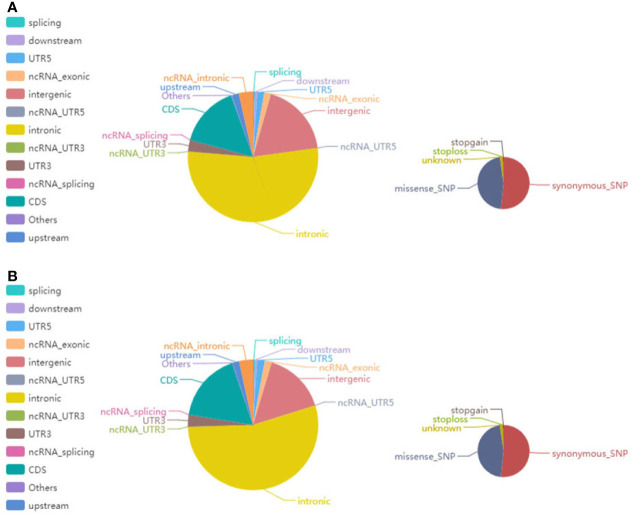
The pie chart shows the distribution of the number of SNPs. **(A)** The left and right images show the number of SNPs in different regions of the genome and the coding regions in tumor tissue, respectively. **(B)** The left and right images reciprocally report the number of SNPs in different regions of the genome and the coding regions in normal tissue.

**Figure 6:**
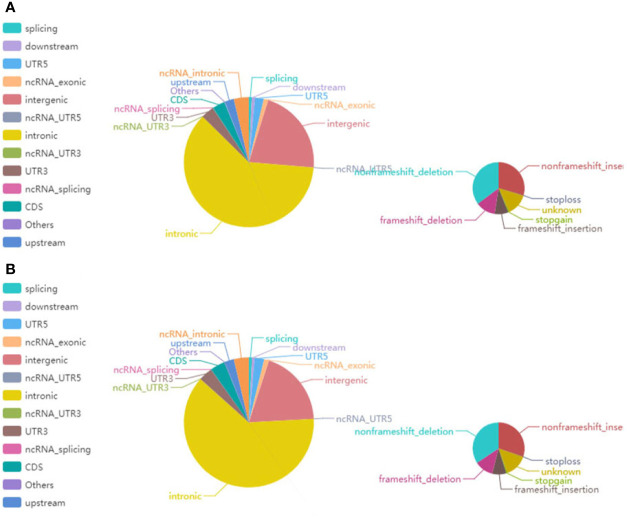
The pie chart represents the distribution of the number of INDELs. **(A)** The left and right images show the number of INDELs on different regions of the genome and in the coding regions in tumor tissue, respectively. **(B)** The left and right images reciprocally report the number of INDELs in different regions of the genome and the coding regions in normal tissue.

In the NGS-based analysis, we sequenced 33,270,383 and 26,042,730 read pairs in the primary renal MALT specimen and adjacent or surrounding normal tissue, respectively. The average proportion of Q30 was more than 80%, and the average error rate was less than 0.1%. Typically, there are approximately 3.6 million SNPs in a person’s genome. Most of the high-frequency SNPs have been recorded in dbSNP. **Supplementary Table 1** shows the number of SNPs in different regions of the genome and coding regions. In primary renal MALT and adjacent specimens, a total of 141,685 and 125,452 SNPs were found and were mainly distributed in intronic, intergenic, and coding sequence (CDS) regions. The number of SNPs in different regions of the genome (left) and the number of SNPs in different types of coding regions (right) are shown in **Figure 5**. The ratio of transformation/transmutation (Ts : Tv) can reflect the accuracy of the SNP dataset. The ratio in primary renal MALT and adjacent specimens was approximately 2.34 and 2.32. Deletions at coding regions or splicing sites may alter protein translation. In addition, one patient harbored a 350K INDEL across the entire genome. The numbers of different INDEL types in the genome and coding regions are shown in **Supplementary Table 2**. There were 18,191 and 15,943 INDELs in the primary renal MALT and adjacent specimens. The number of INDELs in different regions of the genome (left) and the number of INDELs in different types of coding regions (right) are shown in **Figure 6**. We identified 1,908 and 1,619 novel INDELs in the primary renal MALT and adjacent specimens. ANNOVAR software was used to annotate mutation sites.

### Identification of Somatic SNVs, INDELs, and CNVs

**Figure 7:**
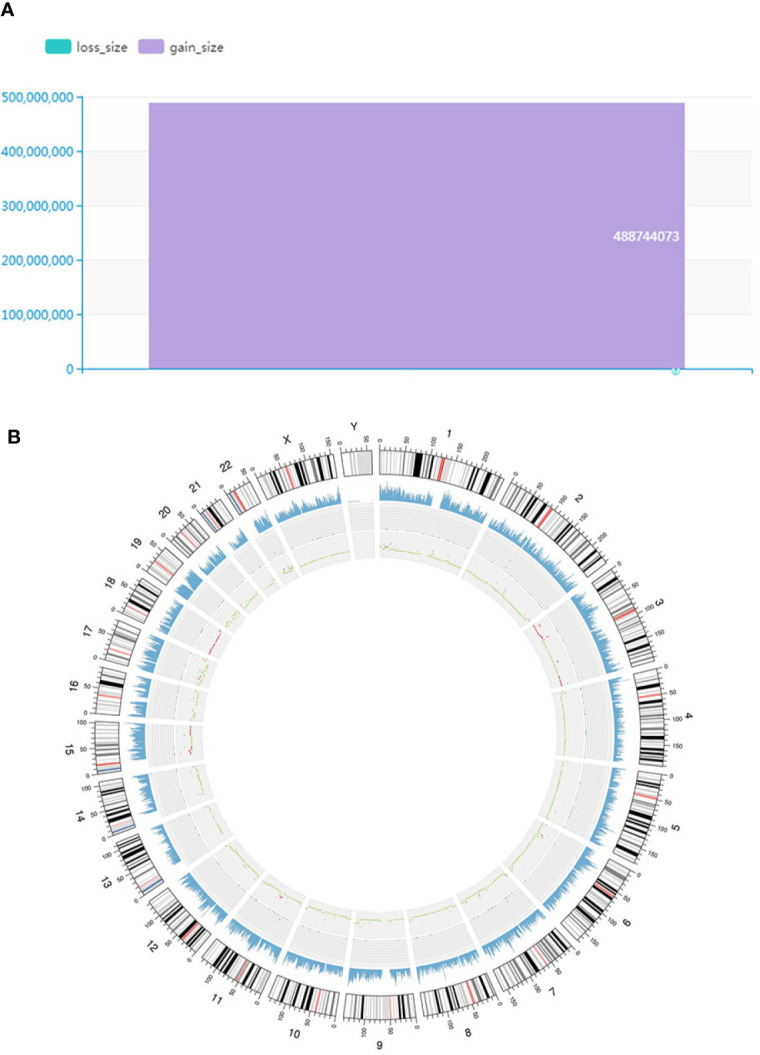
Findings of somatic CNVs distribution and genomic variation analysis. **(A)** Distribution of somatic CNVs. The ordinate represents the total length (mb) of the copy number increase (gain_size) and copy number decrease (loss_size). Furthermore, the abscissa indicates different samples. **(B)** Genomic variation Circos display. The three-layer structure from the outside to the inside represents the sequencing coverage map, the density of SNPs and INDELs and the CNV results, respectively. Red indicates an increased copy number, blue indicates a missing copy number, and green indicates a normal copy number.

An SNV is a variation caused by the substitution of a single nucleotide in the genome. We mainly used MuTect to search for somatic SNV sites. We identified 101 somatic SNVs, and the majority of the identified SNVs were located in CDS and intronic regions. **Supplementary Table 3** shows that the somatic SNVs were located in different regions of the genome. Furthermore, we used Strelka to detect somatic INDEL information and found 7 INDELs; similarly, the somatic INDEL sites were also located in CDS and intronic regions. The somatic INDEL detection results in different regions of the genome are shown in **Supplementary Table 4**. CNVs are represented by the increase or decrease in the copy numbers of genome fragments and are an important part of structural variation (SV), which can be divided into two types: deletion and duplication. Control-FREEC or VarScan software was used to detect somatic CNVs. We identified the gain counts of CNVs as 190 and the total size as 488,744,073 (**Figure 7A**).

### Identification of Predisposing and Driver Mutation Genes

**Figure 8:**
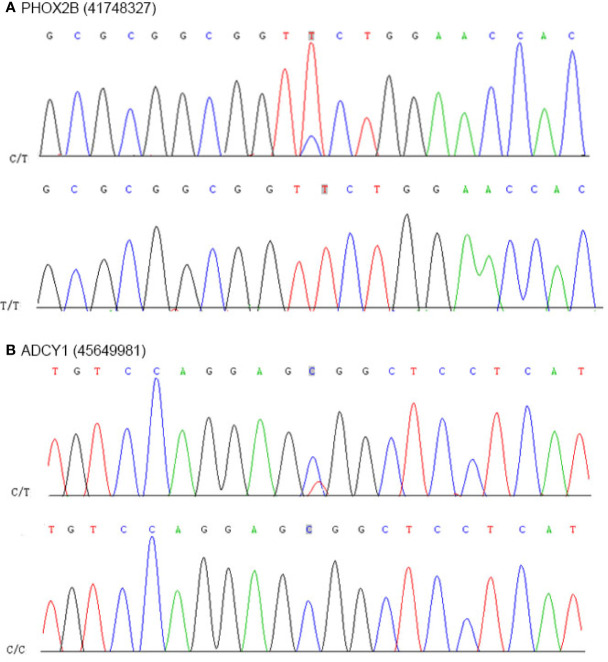
Results of nucleotide Sanger sequencing analysis. **(A)** Sanger sequencing electropherograms of the PHOX2B mutant at position chr19:41748327C>T. **(B)** Sanger sequencing electropherograms of the ADCY1 mutant at position chr19:45649981T>C.

Predisposing genes can be used to encode inherited diseases or to acquire disease susceptibility due to appropriate environmental stimuli. SAMtools software was used to detect germline mutations (SNVs and INDELs), and the results were filtered with the CGC database to screen for possible cancer predisposing genes. The results are shown in **Supplementary Table 5**. Seven predisposing genes (ARID4A, COL2A1, FANCL, ABL2, HSP90AB1, FANCA, and DIS3) were found in one MALT specimen. Furthermore, we compared somatic variation with known driver genes and screened the known driver genes in this tumor sample. Mutations in three driver genes, ACSL3, PHOX2B and ADCY1 were validated. Driver gene analysis results are shown in **Supplementary Table 6**. We used the Circos tool to show somatic cell variation in the MALT sample (**Figure 7B**). Furthermore, we performed secondary confirmation using PCR amplification followed by Sanger sequencing of the DNA fragment containing the mutant base. Our analyses of germline DNA revealed the presence of a mutant base T of PHOX2B and a mutant base C of ADCY1 in the sequence (**Figure 8**).

## Discussion

Kidney primary lymphoma refers to the kidney alone but not systemic lymphoma involving the kidney. Although the kidney is the second most common site of systemic lymphoma involvement, primary lymphoma is rare due to a lack of lymphoid tissue in the kidney (12, 13). Most renal primary lymphomas are high-grade diffuse large B cell lymphomas, whereas low-grade MALT lymphomas are rare. MALT lymphoma is a subtype of marginal lymphoma that often occurs in the stomach, small intestine, lung, parotid gland, etc (5–7). In molecular biology, MALT lymphoma has four types of chromosomal ectopicity: t(11;18)/API2-MALT1 (the most common) and t(1;14)/IgH-BCL10, t(14;18)/IgH-MALT1, and t(3;14)/IgH-FOXP1 (relatively rare). The chromosomal ectopic frequency of MALT lymphomas from different sites is not the same. Due to rare cases of MALT lymphomas in primary kidneys and sporadic case reports, the presence or absence of chromosomal abnormalities requires further study.

After searching the PubMed, Medline, and Scopus databases to identify relevant studies reporting primary MALT lymphoma involving the kidney from inception until 2019, a total of 45 cases of MALT lymphoma involving the kidney were included. Patient-specific and tumor-specific characteristics of reported cases of MALT lymphoma involving the kidney are illustrated in **Supplementary Tables 7** and **8**. MALT lymphoma involving the kidney occurred more often in older men, with a male/female ratio of 26:19. The patients were aged between 9 and 86 years old, with a mean age of 62.5 years. The patients initially presented with gross/microscopic hematuria, severe loin pain anemia, stomachache or hydronephrosis. Due to the latency of symptoms, the course of the disease was often prolonged. In the 45 reported cases, there were 5 patients in the T_1_ stage, 3 in T_2_, and 1 in T_4_, and 35 were unsure. In this report, the patient was in the T_4_ stage. In terms of pathological diagnosis, the immunophenotype analysis showed that the stromal lymphocytes were positive for CD20, CD79a, and BCL-2, while the tumor cells were positive for BCL-10, NF-kB, p65 and PAX-5. Moreover, CD5, CD23, CD10, CD21, BCL-6, and Cyclin D1 were all negative, which could exclude the diagnosis of small lymphocyte lymphoma, follicular lymphoma, and mantle cell lymphoma.

Due to the low prevalence of primary MALT lymphoma in the kidney, there is currently no unified standardized treatment plan. In the 45 reported cases of MALT lymphoma involving the kidney, 11 patients could not be treated surgically, 20 patients were treated with chemoradiotherapy, one patient was treated with antibiotics, and 13 patients underwent surgical treatment, which included seven patients who underwent radical nephrectomy and one patient who underwent radical nephroureterectomy. One patient underwent regional lymphadenectomy, 1 patient underwent kidney cryoablation, and 3 patients underwent an unknown procedure. Therefore, patients with limited lesions and a low clinical stage should be followed up only after surgery. Patients with a high clinical stage and who receive postoperative chemotherapy or radiotherapy may achieve better results. Similar to other sites of MALT lymphoma, the prognosis of patients with primary MALT lymphoma in the kidney is also good. In the 45 reported cases of MALT lymphoma involving the kidney, including the present case, a pooled analysis showed that at a mean follow-up of 35 months, 55.6% of the overall population did not show any evidence of disease, and 4.5% died with the disease. In addition, rituximab, an anti-CD20 monoclonal antibody, has become an effective and well-tolerated first-line treatment for CD20-expressing B-Cell lymphoma due to its ability to bind to CD20 antigens to induce apoptosis and destroy B cells. Rituximab has been shown to have good therapeutic and prognostic effects on MALT (14–16), and it is valuable not only for recurrence, but also in the context of radiotherapy contraindications. Inhibition of the B-Cell receptor (BCR) signaling pathway is becoming a key strategy in B-Cell malignancies. Ibrutinib is a very promising BCR inhibitor, which can irreversibly inactivate BTK (a non-receptor tyrosine kinase in BCR signal). Literature has demonstrated the inhibitory effect of Ibrutinib on *in vitro* cells and its therapeutic potential for B-cell tumors in clinical trials (17).

Regarding the origin of primary MALT lymphoma in the kidney, some scholars believe that lymphoid tissue originates from a kidney cyst, but more scholars believe that chronic inflammatory stimuli cause the renal parenchyma to produce lymphoid tissue, the long-term stimulation of lymphoid tissue continues to proliferate, and then the lesion becomes lymphoma. Some researchers report that patients with primary MALT lymphoma in the kidney have a history of IgA nephropathy, membranous proliferative glomerulonephritis, and pyelonephritis, but some cases have no relevant history (18, 19). The cases described in this article have no history of inflammation. As NGS has a relatively quick turnaround, low costs, and high coverage, mutations in two driver genes, ACSL3 and PHOX2B, were validated. Because it is highly expressed in the brain and testis and weakly expressed in adipose tissue and the kidney, ACSL3 can participate in the synthesis of saturated fatty acids, including laurate and myristate, or unsaturated fatty acids, including arachidonate and eicosapentaenoate. The literature has reported that the expression of ACSL3 is significantly higher in metastatic prostate tumors than in primary prostate tumors (20–22). Encoding a paired-like homeobox domain transcription factor in the central and peripheral autonomic nervous systems, PHOX2B, which regulates the differentiation and survival of neurons, is located on chromosome 4p12. As one of the major disease-causing genes in central hypoventilation syndrome, germline mutations in PHOX2B were first found in neuroblastoma patients (23, 24).

Furthermore, we also found several predisposing genes, including ARID4A, COL2A1, DIS3, and FANCA, in MALT specimens. The ARID1A-encoded protein, a ubiquitously expressed nuclear protein, binds to retinoblastoma protein and hence regulates cell proliferation. ARID4A has been considered a tumor suppressor gene because it is frequently mutated or weakly expressed in breast, oral, tongue, and colorectal cancers and leukemia (25–27). The alpha 1 chain of type II collagen, which is encoded by COL2A1, is the major collagen constituent of articular cartilage. Some studies have reported that the COL2A1 mutation in high-grade cartilaginous tumors is significantly higher than that in low-grade cartilaginous tumors (28, 29). DIS3, an active part of the human exosome complex, has exo- and endoribonucleolytic activity that promotes kinetochore formation and forms interactions in kinetochore-microtubules. DIS3 mutations have been found in some patients with multiple myeloma (MM), and these patients have a lower median overall survival than other MM patients (30, 31). The Fanconi anemia complementation group A (FANCA) gene is located at the telomere of chromosome 16 and is mutated in two-thirds of all Fanconi anemia (FA) cases due to the abundance of Alu elements (32). Because FANCA plays an important role in the repair of DNA damage, it is considered a potential tumor suppressor gene (33). In addition, SNPs in FANCA are associated with an 8% increase in breast cancer risk (34).

In summary, we performed whole-exome sequencing based tumor mutation profiling for a 77-year-old female presenting with discomfort at the waist was pathologically diagnosed as MALT lymphomas in the right kidney. With respect to genetic investigation, seven predisposing genes including ARID4A, COL2A1, FANCL, ABL2, HSP90AB1, FANCA, and DIS3 were found in renal MALT specimen. Sanger sequencing of germline DNA revealed the presence of a mutant base T of PHOX2B and a mutant base C of ADCY1 in the sequence, which were discovered for the first time in MALT lymphomas involving the kidney. Moreover, immunohistochemical analysis revealed that tumor cells were positive for CD20, CD79a, PAX5, CD21, and CD23 and expression of CD3, CD5, and CD8 were observed in reactive T lymphocytes surrounding tumor cells. We also summarized the patient-specific and tumor-specific characteristics of reported cases of MALT lymphoma involving the kidney and investigated the clinical history, diagnostic method, treatment, prognosis monitoring, and pathological evaluation of MALT lymphomas in the kidney. However, our review also has limitations. First, only a very limited number of cases were available for analysis because of the rarity of the disease, and the investigation spans approximately 20 years, which may be scant to provide meaningful statistical results and need multicenter research to investigate clinical profile for MALT lymphomas in the kidney. Second, although we analyzed potential driver mutations (ACSL3, PHOX2B and ADCY1) by whole-genome sequencing, the precise mechanism about passenger mutations or functional data warrants further investigation based on primary cell culture technology from tissues of MALT lymphomas in the kidney, which may be beneficial to evaluate genetic alterations. Meanwhile, as our samples for whole-genome sequencing were limited, it is hard to avoid false discovery rates related to multiple testing effects completely, indicating that our conclusions of hypothesis need to be elaborated or explained carefully.

## Data Availability Statement

The data presented in the study are deposited in the NCBI database SRA repository, accession number: SRR11038941 and SRR11038942 (http://www.ncbi.nlm.nih.gov/sra/?term=SRR11038941 and https://www.ncbi.nlm.nih.gov/sra/?term=SRR11038942).

## Ethics Statement

The studies involving human participants were reviewed and approved by Regional Ethics Committee of Dalian Friendship Hospital. The patients/participants provided their written informed consent to participate in this study.

## Author Contributions 

SW, TL, and HZ collected and analyzed the patient’s clinical data and designed the research. XZ and HJ performed and interpreted pathology staining. MS, ZY, and HL performed the review of literature and drafted the manuscript. BF, SW, TL, ZN, and RZ performed molecular analyses. BF and HZ assisted with image interpretation and formatting and critically revised the manuscript. All authors contributed to the article and approved the submitted version.

## Funding

The present study was supported by the National Natural Science Foundation of China (grant no. 31800787), the Natural Science Foundation of Liaoning Province (grant no. LQ2017025), the Doctoral Research Startup Foundation of Liaoning Province (grant no. 20180540020), and the Medical Scientific Research Project of Dalian City (grant no. 1812038), the United Fund of the Second Hospital of Dalian Medical University and Dalian Institute of Chemical Physics, Chinese Academy of Sciences (UF-QN-202004), the Dalian High-level Talents Innovation Support Program (2019RQ014).

## Conflict of Interest

The authors declare that the research was conducted in the absence of any commercial or financial relationships that could be construed as a potential conflict of interest.
