# Supplementary material for: Whole-Exome Sequencing Reveals New Potential Mutations Genes for Primary Mucosa-Associated Lymphoid Tissue Lymphoma Arising From the Kidney
Source: Front Oncol. 2021 Jan 8;10:609839. doi: 10.3389/fonc.2020.609839 (PMC7873889; doi:10.3389/fonc.2020.609839)
Supplement: Supplementary file 1 [file DataSheet_1.docx]

Supplementary Material

# Supplementary Tables

Supplementary table 1. The number of SNPs in different regions of the genome and in coding regions.

| Sample | Primay tumor | Normal |
| --- | --- | --- |
| CDS | 21,755 | 21,648 |
| Synonymous_SNP | 11,229 | 11,201 |
| Missense_SNP | 10,033 | 9,956 |
| Stopgain | 72 | 68 |
| Stoploss | 9 | 9 |
| Unknown | 412 | 414 |
| Intronic | 75,788 | 68,192 |
| UTR3 | 4,044 | 3,726 |
| UTR5 | 2,461 | 2,380 |
| Splicing | 508 | 502 |
| ncRNA_exonic | 2,373 | 2,214 |
| ncRNA_intronic | 5,018 | 4,335 |
| ncRNA_UTR3 | 0 | 0 |
| ncRNA_UTR5 | 0 | 0 |
| ncRNA_splicing | 24 | 23 |
| Upstream | 2,554 | 2,074 |
| Downstream | 953 | 772 |
| Intergenic | 26,036 | 19,442 |
| Others | 171 | 144 |
| Total | 141,685 | 125,452 |

Supplementary table 2. The number of INDELs in different regions of the genome and in coding regions.

| Sample | Primay tumor | Normal |
| --- | --- | --- |
| CDS | 21,755 | 21,648 |
| Synonymous_SNP | 11,229 | 11,201 |
| Missense_SNP | 10,033 | 9,956 |
| Stopgain | 72 | 68 |
| Stoploss | 9 | 9 |
| Unknown | 412 | 414 |
| Intronic | 75,788 | 68,192 |
| UTR3 | 4,044 | 3,726 |
| UTR5 | 2,461 | 2,380 |
| Splicing | 508 | 502 |
| ncRNA_exonic | 2,373 | 2,214 |
| ncRNA_intronic | 5,018 | 4,335 |
| ncRNA_UTR3 | 0 | 0 |
| ncRNA_UTR5 | 0 | 0 |
| ncRNA_splicing | 24 | 23 |
| Upstream | 2,554 | 2,074 |
| Downstream | 953 | 772 |
| Intergenic | 26,036 | 19,442 |
| Others | 171 | 144 |
| Total | 141,685 | 125,452 |

Supplementary table 3. The number of Somatic SNVs in different regions of the genome.

| Sample | Primary tumor |
| --- | --- |
| CDS | 38 |
| Synonymous_SNP | 10 |
| Missense_SNP | 26 |
| Stopgain | 1 |
| Stoploss | 0 |
| Unknown | 1 |
| Intronic | 31 |
| UTR3 | 1 |
| UTR5 | 1 |
| Splicing | 1 |
| ncRNA_exonic | 3 |
| ncRNA_intronic | 2 |
| ncRNA_UTR3 | 0 |
| ncRNA_UTR5 | 0 |
| ncRNA_splicing | 0 |
| Upstream | 1 |
| Downstream | 0 |
| Intergenic | 23 |
| Others | 0 |
| Total | 101 |

Supplementary table 4. The number of Somatic INDELs in different regions of the genome.

| Sample | Primary tumor |
| --- | --- |
| CDS | 5 |
| Frameshift_deletion | 3 |
| Frameshift_insertion | 1 |
| Nonframeshift_deletion | 0 |
| Nonframeshift_insertion | 1 |
| Stopgain | 0 |
| Stoploss | 0 |
| Unknown | 0 |
| intronic | 2 |
| UTR3 | 0 |
| UTR5 | 0 |
| Splicing | 0 |
| ncRNA_exonic | 0 |
| ncRNA_intronic | 0 |
| ncRNA_UTR3 | 0 |
| ncRNA_UTR5 | 0 |
| ncRNA_splicing | 0 |
| Upstream | 0 |
| Downstream | 0 |
| Intergenic | 0 |
| Others | 0 |
| Total | 7 |

Supplementary table 5. Analysis results of predisposing genes.

| Hugo_Symbol | Chromosome | Position | Ref_allele | Alt_allele | Variant_Classification | AAChange |
| --- | --- | --- | --- | --- | --- | --- |
| ARID4A | 14 | 58813201 | A | T | Missense_Mutation | ARID4A:NM_002892:exon13:c.A1052T:p.Y351F\|ARID4A:NM_023000:exon13:c.A1052T:p.Y351F\|ARID4A:NM_023001:exon13:c.A1052T:p.Y351F |
| COL2A1 | 12 | 48371135 | G | A | Missense_Mutation | COL2A1:NM_033150:exon45:c.C3034T:p.P1012S\|COL2A1:NM_001844:exon46:c.C3241T:p.P1081S |
| FANCL | 2 | 58392889 | G | A | Missense_Mutation | FANCL:NM_001114636:exon8:c.C676T:p.R226W\|FANCL:NM_018062:exon8:c.C661T:p.R221W |
| ABL2 | 1 | 1.79E+08 | C | T | Missense_Mutation | ABL2:NM_001136001:exon3:c.G341A:p.R114Q\|ABL2:NM_001168236:exon3:c.G341A:p.R114Q\|ABL2:NM_001168238:exon3:c.G341A:p.R114Q\|ABL2:NM_001168239:exon3:c.G296A:p.R99Q\|ABL2:NM_001136000:exon4:c.G359A:p.R120Q\|ABL2:NM_001168237:exon4:c.G404A:p.R135Q\|ABL2:NM_005158:exon4:c.G359A:p.R120Q\|ABL2:NM_007314:exon4:c.G404A:p.R135Q |
| HSP90AB1 | 6 | 44220960 | C | T | Missense_Mutation | HSP90AB1:NM_001271969:exon11:c.C1910T:p.T637M\|HSP90AB1:NM_001271970:exon11:c.C1910T:p.T637M\|HSP90AB1:NM_001271971:exon11:c.C1766T:p.T589M\|HSP90AB1:NM_001271972:exon11:c.C1880T:p.T627M\|HSP90AB1:NM_007355:exon11:c.C1910T:p.T637M |
| FANCA | 16 | 89849425 | G | A | Missense_Mutation | FANCA:NM_000135:exon16:c.C1556T:p.A519V\|FANCA:NM_001286167:exon16:c.C1556T:p.A519V |
| DIS3 | 13 | 73355136 | A | T | Missense_Mutation | DIS3:NM_014953:exon2:c.T234A:p.D78E |

Supplementary table 6. Analysis results of driving genes

| Hugo_Symbol | Chromosome | Position | Ref_allele | Alt_allele | Variant_  Classification | AAChange | CGC | Comprehensive435 |
| --- | --- | --- | --- | --- | --- | --- | --- | --- |
| ACSL3 | 2 | 223,795,341 | G | A | Missense_Mutation | ACSL3:NM_203372:exon13:c.G1543A:p.G515R\|ACSL3:NM_004457:exon14:c.G1543A:p.G515R | prostate | - |
| PHOX2B | 4 | 41,748,327 | T | C | Missense_Mutation | PHOX2B:NM_003924:exon3:c.A442G:p.N148D | neuroblastomaneuroblastoma | - |
| ADCY1 | 7 | 45,649,981 | C | T | Missense_Mutation | ADCY1:NM_021116:exon3:c.C793T:p.R265W\|ADCY1:NM_001281768:exon4:c.C118T:p.R40W | - | High Confidence Driver |

Supplementary table 7. Patient-specific characteristics of reported cases of MALT Lymphoma Involving the Kidney

| Author | Sex | Age at diagnosis | History of Lymphoma | Presentation | Associated  Disease | Treatment | Follow-up  (month) | Outcome |
| --- | --- | --- | --- | --- | --- | --- | --- | --- |
| Li SJ  (2014)（1） | M | 52 | No | Anemia, Stomachache | MPGN | Radiotherapy andSteroids | 12 | Alive |
| Niwa N  (2014)（2） | F | 86 | No | Not available | Urinary tract infection | Radical nephroureterectomy | 4 | No evidence of disease |
| [Asgari SA](https://www.ncbi.nlm.nih.gov/pubmed/?term=Asgari SA[Author]&cauthor=true&cauthor_uid=24678358)  (2014)（3） | F | 74 | No | Gross hematuria  Right ﬂank pain | Right kidney  Stone | Not available | - | Unknown |
| Vedovo F  (2014)（4） | F | 82 | No | Not available | None | Radical nephrectomy  + lymph node dissection, + Chemotherapy  (rituximab) | 10 | No evidence of disease |
| [Chi PJ](https://www.ncbi.nlm.nih.gov/pubmed/?term=Chi PJ[Author]&cauthor=true&cauthor_uid=24685302)  (2014)（5） | F | 72 | No | Asymptomatic | Chronic kidney disease | Chemotherapy  (Leukeran + prednisolone) | - | No evidence of disease |
| Jaso J  (2012)（6） | M | 74 | No | Not available | None | Chemotherapy  (Rituximab + cyclophosphamide) | 114 | Alive with progressive disease |
| Kasmani R  (2011)（7） | M | 58 | No | Lower extremity swelling;  Scrotal edema; Abdominal distension | None | Chemotherapy | - | Unknown |
| Charitaki E (2011)（8） | F | 66 | No | Acute kidney injury, hypertension | None | Chemotherapy  (R-CHOP) +  Kidney cryoablation | 36 | No evidence of disease |
| [Schniederjan SD](https://www.ncbi.nlm.nih.gov/pubmed/?term=Schniederjan SD[Author]&cauthor=true&cauthor_uid=19377442)  (2009)（9） | F | 54 | No | Not available | None | Not available | 24 | No evidence of disease |
|  | M | 65 | No | Not available | None | Not available | - | Unknown |
|  | M | 75 | No | Not available | None | Not available | - | Unknown |
|  | F | 50 | No | Not available | None | Not available | 24 | No evidence of disease |
| Kato Y  (2008)（10） | M | 30 | No | Frequent urination ,  Right lower abdominal pain | Nephrotic syndrome | Radical nephrectomy | 21 | No evidence of disease |
| [Garcia M](https://www.ncbi.nlm.nih.gov/pubmed/?term=Garcia M[Author]&cauthor=true&cauthor_uid=17709321)  （2007）（11） | F | 77 | No | Not available | None | Not available | 36 | No evidence of disease |
|  | M | 65 | No | Not available | Renal actinomycosis | Antibiotics | 14 | No evidence of disease |
|  | M | 54 | No | Not available | None | Chemotherapy | 36 | No evidence of disease |
|  | F | 75 | No | Not available | Bladder cancer | Nephrectomy  + Chemotherapy | 12 | No evidence of disease |
|  | M | 66 | No | Not available | None | Nephrectomy | 53 | No evidence of disease |
|  | M | 83 | No | Not available | None | Chemotherapy | 9 | No evidence of disease |
|  | F | 65 | No | Not available | Helicobacter pylori gastritis | Nephrectomy | 6 | Alive, retroperitoneal lymphadenopathy |
|  | M | 73 | MALT lymphoma, ocular adnexa (8 year earlier) | Not available | Colon adenocarcinoma | Chemotherapy | 9 | Alive, residual renal mass |
|  | M | 47 | No | Not available | None | Chemotherapy + autologous stem cell transplantation | 42 | No evidence of disease |
|  | F | 18 | MALT lymphoma, parotid gland and cervical lymph node | Not available | Helicobacter pylori gastritis | Chemotherapy | 10 | No evidence of disease |
| Qiu L  (2006)（12） | F | 83 | No | Back pain, Abdominal bloating | None | Chemotherapy | 8 | No evidence of disease |
|  | M | 53 | No | Asymptomatic | None | Partial nephrectomy | 10 | No evidence of disease |
|  | M | 72 | No | Fever;  Chills, Abdominal pain, Weight loss | None | Radical nephrectomy | - | Unknown |
| Mortlock AM  (2006)（13） | F | 84 | No | Painless swelling of legs;  Shortness of breath;  Weight loss | Systemic lupus erythematosus | Chemotherapy |  | No evidence of disease |
| [Tuzel E](https://www.ncbi.nlm.nih.gov/pubmed/?term=Tuzel E[Author]&cauthor=true&cauthor_uid=12597979)  (2003)（14） | M | 43 | No | Left flank pain | None | Nephrectomy | 28 | No evidence of disease |
| [Stokes MB](https://www.ncbi.nlm.nih.gov/pubmed/?term=Stokes MB[Author]&cauthor=true&cauthor_uid=12005247)  (2002)（15） | M | 68 | No | Fatigue;  Malaise | MPGN Hypertensive | Prednisone |  | Not available |
|  | F | 72 | No | Progressive renal failure; Peripheral edema | MPGN | Prednisone | 6 | No evidence of disease |
| [Jindal B](https://www.ncbi.nlm.nih.gov/pubmed/?term=Jindal%20B%5bAuthor%5d&cauthor=true&cauthor_uid=11464127)  (2001)（16） | M | 45 | No | Fever ;  Abdominal pain | None | Chemotherapy  (CHOP) | 156 | Alive, with multiple relapses |
| Tao J  ( 2000)（17） | M | 9 | No | Not available | EBV infection | Chemotherapy | 48 | Died with lymphoma |
| [Colović M](https://www.ncbi.nlm.nih.gov/pubmed/?term=Colovi%C4%87 M[Author]&cauthor=true&cauthor_uid=10651124)  (1999)（18） | M | 50 | No | Not available | Helicobacter pylori gastritis | Nephrectomy | - | Not available |
| [Mhawech P](https://www.ncbi.nlm.nih.gov/pubmed/?term=Mhawech P[Author]&cauthor=true&cauthor_uid=10835539)  (1999)（18） | M | 76 | No | Right flank pain; Anorexia;  Weight loss | Essential hypertension |  |  |  |
| Anderson CM  (1998)（19） | M | 45 | No | Not available | None | Not available | 28.8 | No evidence of disease |
|  | F | 77 | No | Not available | None | Not available | 48 | No evidence of disease |
| Mak SK  (1998)（20） | M | 62 | No | Not available | IgA nephropathy | Chemotherapy | 20 | No evidence of disease |
| Araki K  (1998)（21） | M | 68 | No | Not available | None | No | 60 | Alive with disease |
| Anderson CM  (1998)（21） | M | 45 | No | Not available | None | Not available | 28.8 | No evidence of disease |
|  | F | 77 | No | Not available | None | Not available | 48 | No evidence of disease |
| Imahori SC  (1994)（22） | M | 56 | No | Not available | None | Nephrectomy | 156 | Died with lymphoma |
| Parveen T  (1993)（23） | F | 69 | No | Not available | None | Radical nephrectomy,  irradiation | 17 | No evidence of disease |
| [Pelstring RJ](https://www.ncbi.nlm.nih.gov/pubmed/?term=Pelstring RJ[Author]&cauthor=true&cauthor_uid=1746490)  (1991)（24） | F | 62 | No | Not available | Sjögren syndrome | Chemotherapy | 15 | Alive with splenomegaly |
| This case | F | 77 | No | Right flank pain  Gross hematuria | None | Radical nephrectomy | 49 | No evidence of disease |

MPGN, Membrane proliferative glomerulonephritis; CHOP cyclophosphamide/doxorubicin/vincristine/prednisone

Supplementary table 8. Tumor-specific characteristics of reported cases of MALT Lymphoma Involving the Kidney

| Author | Tumor  side | Extrarenal | Tumor  size (cm) | T  Stage | Immunohistochemistry | |
| --- | --- | --- | --- | --- | --- | --- |
|  |  | Location |  |  | Tumor cells | Stromal lymphocytes |
| Niwa N  (2014)（2） | Right | No | Unknown | Unknown | - | CD20, CD79a |
| Vedovo F  (2014)（4） | Left | No | 7.0×3.2×3.4 | T_1_ | - | CD138, CD20, CD3, CD43 |
| [Asgari SA](https://www.ncbi.nlm.nih.gov/pubmed/?term=Asgari%20SA%5bAuthor%5d&cauthor=true&cauthor_uid=24678358)  (2014)（3） | Right | Ipsilateral ureter | 6.2×4.2×3.7 | T_1_ | Bcl-2 | CD5, CD20 |
| [Chi PJ](https://www.ncbi.nlm.nih.gov/pubmed/?term=Chi PJ[Author]&cauthor=true&cauthor_uid=24685302)  (2014)（5） | Right | Fibrovascular tissue | 6.4×5.6 | T_1_ | - | CD20 |
| Jaso J  (2012)（6） | Unknown | Retroperitoneum Chest wall | Unknown | T_4_ | - | CD5, CD19, CD20, CD38, FMC-7 |
| Kasmani R  (2011)（7） | Unknown | Abdomen and pelvis | Unknown | Unknown | - | CD19, CD20,CD22 |
| Charitaki E  (2011)（8） | Unknown | No | Unknown | Unknown | - | CD45,CD20,CD79a |
| Kato Y  (2008)（10） | Right | No | 4.0×2.5×2.7 | T_1_ | - | CD20, CD79a |
| [Garcia M](https://www.ncbi.nlm.nih.gov/pubmed/?term=Garcia%20M%5bAuthor%5d&cauthor=true&cauthor_uid=17709321)  （2007）（11） | Unknown | No | Unknown | Unknown | Bcl-10, NF-kB p65 | CD20 |
|  | Right | No | Unknown | Unknown | - | CD20 |
|  | Left | No | Unknown | Unknown | Bcl-10, NF-kB p65 | CD20 |
|  | Right | No | Unknown | Unknown | Bcl-10, NF-kB p65 | CD20 |
|  | Left | No | Unknown | Unknown | Bcl-10, NF-kB p65 | CD20 |
|  | Right | No | Unknown | Unknown | - | CD20 |
|  | Left | Retroperitoneal  lymph nodes | Unknown | Unknown | Bcl-10, NF-kB p65 | CD20 |
|  | Left | No | Unknown | Unknown | - | CD20 |
|  | Left | Soft tissue of  Flank and thigh | Unknown | Unknown | - | CD20 |
|  | Right | No | Unknown | Unknown | - | CD20 |
| Mortlock AM  (2006)（13） | Right | Right para-aortic lymph nodes | Unknown | Unknown | Bcl-2 | CD20 |
| Qiu L  (2006)（12） | Left | Renal pelvis,  sinus, left proximal ureter | Unknown | Unknown | - | CD20,CD79a, Bcl-2 |
| [Tuzel E](https://www.ncbi.nlm.nih.gov/pubmed/?term=Tuzel E[Author]&cauthor=true&cauthor_uid=12597979)  (2003)（14） | Right | No | 9×5 | T_2_ |  | CD20, CD3, CD43 |
| [Stokes MB](https://www.ncbi.nlm.nih.gov/pubmed/?term=Stokes MB[Author]&cauthor=true&cauthor_uid=12005247)  (2002)（15） | Unknown | Perinephric adipose tissue. |  |  |  | CD20 |
| [Jindal B](https://www.ncbi.nlm.nih.gov/pubmed/?term=Jindal B[Author]&cauthor=true&cauthor_uid=11464127)  (2001)（16） | Left | No | 4×3×3 | Unknown | - | CD20, CD45RO |
| Tao J  ( 2000)（17） | Right | Axillary lymph node, lung | Unknown | Unknown | - | CD19,CD20,IgG，K |
| [Mhawech P](https://www.ncbi.nlm.nih.gov/pubmed/?term=Mhawech%20P%5bAuthor%5d&cauthor=true&cauthor_uid=10835539)  (2000)（25） | Right | No | 5.0×3.0×3.2 |  | - | CD20 |
| [Colović M](https://www.ncbi.nlm.nih.gov/pubmed/?term=Colovi%C4%87%20M%5bAuthor%5d&cauthor=true&cauthor_uid=10651124)  (1999)（18） | Right | Psoas muscle | 6.0×4.5×5.8 | Unknown | - | CD20, CD79α, CD43 |
|  | Left | No |  |  |  | CD20 |
|  | Left | No | 2.5×1.9 | T_1_ | - | CD20,CD79a |
|  | Right | Orbit | 7.5 | T_2_ | - | CD20,CD79a |
| Mak SK  (1998)（20） | Unknown | GI tract; tonsils | Unknown | Unknown | - | Unknown |
| Araki K  (1998)（21） | Bilateral | Prostate, salivary glands | Unknown | Unknown | - | Unknown |
| Imahori SC  (1994)（22） | Left | Orbit | Unknown | Unknown | - | Unknown |
| Parveen T  (1993)（23） | Right | Perirenal lymph nodes | Unknown | Unknown | - | Unknown |
| [Pelstring RJ](https://www.ncbi.nlm.nih.gov/pubmed/?term=Pelstring%20RJ%5bAuthor%5d&cauthor=true&cauthor_uid=1746490)  (1991)（24） | Unknown | Parotid gland | Unknown | Unknown | Unknown | Unknown |
| This case | Right | No | 6.0x5.5x4.2 |  | Ki-67, Bcl-2,  PAX-5, Bcl-6 | CD3, CD5 , CD8,  CD20,CD79a |

EMA: Epithelial membrane antigen; CK: cytokeratin;

**REFERENCES**

1. Li SJ, Chen HP, Chen YH, Zhang LH, Tu YM, Liu ZH. Renal involvement in non-Hodgkin lymphoma: proven by renal biopsy. *PLoS One.* （2014） 9:e95190. doi:10.1371/journal.pone.0095190

2. Niwa N, Tanaka N, Horinaga M, Hongo H, Ito Y, Watanabe T, et al. Mucosa-associated lymphoid tissue lymphoma arising from the kidney. *Can Urol Assoc J.* （2014） 8:E86-8. doi:10.5489/cuaj.1533

3. Asgari SA, Aval HB, Asgari SA, Kheradmand K. A unique case of kidney's collecting system MALT lymphoma. *Can Urol Assoc J.* （2014） 8:E172-5. doi:10.5489/cuaj.1452

4. Vedovo F, Pavan N, Liguori G, Bussani R, Siracusano S, Trombetta C. [Incidence and distribution of lymphoid neoplasm of the urinary tract and male genital organs in an urban area of northern Italy in the last decade]. *Urologia.* （2014） 81 Suppl 23:S20-3. doi:10.5301/RU.2014.11981

5. Chi PJ, Pei SN, Huang TL, Huang SC, Ng HY, Lee CT. Renal MALT lymphoma associated with Waldenström macroglobulinemia. *J Formos Med Assoc.* （2014） 113:255-7. doi:10.1016/j.jfma.2011.02.007

6. Jaso J, Chen L, Li S, Lin P, Chen W, Miranda RN, et al. CD5-positive mucosa-associated lymphoid tissue (MALT) lymphoma: a clinicopathologic study of 14 cases. *Hum Pathol.* （2012） 43:1436-43. doi:10.1016/j.humpath.2011.11.004

7. Kasmani R, Marina VP, Abidi S, Johar B, Malhotra D. Minimal change disease associated with MALT lymphoma. *Int Urol Nephrol.* （2012） 44:1911-3. doi:10.1007/s11255-011-9992-z

8. Charitaki E, Liapis K, Moutzouris DA, Marinos L, Adamidis K, Margellos V, et al. Primary renal MALT lymphoma presenting with cryoglobulinaemia. *Nephrol Dial Transplant.* （2011） 26:3819-21. doi:10.1093/ndt/gfr478

9. Schniederjan SD, Osunkoya AO. Lymphoid neoplasms of the urinary tract and male genital organs: a clinicopathological study of 40 cases. *Mod Pathol.* （2009） 22:1057-65. doi:10.1038/modpathol.2009.65

10. Kato Y, Hasegawa M, Numasato S, Monma N, Fujioka T. Primary mucosa-associated lymphoid tissue-type lymphoma arising in the kidney. *Int J Urol.* （2008） 15:90-2. doi:10.1111/j.1442-2042.2007.01923.x

11. Garcia M, Konoplev S, Morosan C, Abruzzo LV, Bueso-Ramos CE, Medeiros LJ. MALT lymphoma involving the kidney: a report of 10 cases and review of the literature. *Am J Clin Pathol.* （2007） 128:464-73. doi:10.1309/0T2UKUKV91W3QR6W

12. Qiu L, Unger PD, Dillon RW, Strauchen JA. Low-grade mucosa-associated lymphoid tissue lymphoma involving the kidney: report of 3 cases and review of the literature. *Arch Pathol Lab Med.* （2006） 130:86-9. doi:10.1043/1543-2165(2006)130[86:LMLTLI]2.0.CO;2

13. Mortlock AM, Lim CS, Morgan H, Wong TW, Joshi A, Kuttikat A, et al. Renal MALToma: an unusual lymphoma in a patient with lupus. *Lupus.* （2006） 15:613-5. doi:10.1177/0961203306071920

14. Tuzel E, Mungan MU, Yorukoglu K, Basakci A, Kirkali Z. Primary renal lymphoma of mucosa-associated lymphoid tissue. *Urology.* （2003） 61:463. doi:10.1016/s0090-4295(02)02267-7

15. Stokes MB, Wood B, ChE A. Membranoproliferative glomerulonephritis associated with low-grade B cell lymphoma presenting in the kidney. *Clin Nephrol.* （2002） 57:303-9. doi:10.5414/cnp57303

16. Jindal B, Sharma SC, Das A, Banerjee AK. Indolent behaviour of low-grade B cell lymphoma of mucosa-associated lymphoid tissue arising in the kidney. *Urol Int.* （2001） 67:91-3. doi:10.1159/000050955

17. Tao J, Kahn L. Epstein-Barr virus-associated high-grade B-cell lymphoma of mucosal-associated lymphoid tissue in a 9-year-old Boy. *Arch Pathol Lab Med.* （2000） 124:1520-4. doi:10.1043/0003-9985(2000)124<1520:EBVAHG>2.0.CO;2

18. Colović M, Hadzi-Djokić J, Cemerikić V, Colović R, Janković G, Dacić M. Primary MALT lymphoma of the kidney. *Hematol Cell Ther.* （1999） 41:229-32. doi:10.1007/s00282-999-0229-x

19. Anderson CM, Pusztai L, Palmer JL, Cabanillas F, Ellerhorst JA. Coincident renal cell carcinoma and nonHodgkin's lymphoma: the M. D. Anderson experience and review of the literature. *J Urol.* （1998） 159:714-7.

20. Mak SK, Wong PN, Lo KY, Wong AK. Successful treatment of IgA nephropathy in association with low-grade B-cell lymphoma of the mucosa-associated lymphoid tissue type. *Am J Kidney Dis.* （1998） 31:713-8. doi:10.1053/ajkd.1998.v31.pm9531192

21. Araki K, Kubota Y, Iijima Y, Suzuki H, Sasagawa I, Nakada T, et al. Indolent behaviour of low-grade B-cell lymphoma of mucosa-associated lymphoid tissue involved in salivary glands, renal sinus and prostate. *Scand J Urol Nephrol.* （1998） 32:234-6. doi:10.1080/003655998750015665

22. Imahori SC. Low-grade B-cell lymphoma of mucosa-associated lymphoid tissue involving kidney. *Arch Pathol Lab Med.* （1994） 118:111-2.

23. Parveen T, Navarro-Roman L, Medeiros LJ, Raffeld M, Jaffe ES. Low-grade B-cell lymphoma of mucosa-associated lymphoid tissue arising in the kidney. *Arch Pathol Lab Med.* （1993） 117:780-3.

24. Pelstring RJ, Essell JH, Kurtin PJ, Cohen AR, Banks PM. Diversity of organ site involvement among malignant lymphomas of mucosa-associated tissues. *Am J Clin Pathol.* （1991） 96:738-45. doi:10.1093/ajcp/96.6.738

25. Mhawech P, Ahearn J, Medeiros LJ. Pathologic quiz case. A unilateral renal mass in an elderly woman. *Arch Pathol Lab Med.* （2000） 124:919-20. doi:10.1043/0003-9985(2000)124<0919:PQCAUR>2.0.CO;2
